# Supplementary material for: Environmental stimuli shape microglial plasticity in glioma
Source: eLife. 2017 Dec 29;6:e33415. doi: 10.7554/eLife.33415 (PMC5774898; doi:10.7554/eLife.33415)
Supplement: Figure 1—source data 1. — Raw data of Gene Ontology over-representation analysis (Bonferroni corrected p-values <0.05) of the genes that are upregulated in microglia after EE exposure.Image: raw data refer to Figure 1 data. [file elife-33415-fig1-data1.docx]

| \| **Gene Ontology term** \| **P - values** \| \| --- \| --- \| \| response to interferon-gamma \| 1,70E-19 \| \| innate immune response \| 6,90E-18 \| \| response to cytokine \| 1,10E-17 \| \| cellular response to cytokine stimulus \| 9,80E-14 \| \| response to other organism \| 5,70E-12 \| \| response to bacterium \| 1,20E-11 \| \| positive regulation of immune system process \| 1,90E-09 \| \| cytokine-mediated signaling pathway \| 3,60E-09 \| \| adaptive immune response based on somatic recombination of immune receptors built from immunoglobulin superfamily domains \| 4,40E-09 \| \| response to molecule of bacterial origin \| 9,90E-09 \| \| defense response to other organism \| 1,20E-08 \| \| cellular response to lipopolysaccharide \| 2,20E-08 \| \| cellular response to molecule of bacterial origin \| 3,70E-08 \| \| response to lipopolysaccharide \| 1,00E-07 \| \| positive regulation of T cell activation \| 2,20E-07 \| \| positive regulation of homotypic cell-cell adhesion \| 2,70E-07 \| \| positive regulation of leukocyte cell-cell adhesion \| 2,70E-07 \| \| regulation of T cell activation \| 2,80E-07 \| \| regulation of leukocyte cell-cell adhesion \| 3,60E-07 \| \| cellular response to organic substance \| 4,10E-07 \| \| negative regulation of immune system process \| 4,20E-07 \| \| regulation of homotypic cell-cell adhesion \| 5,40E-07 \| \| positive regulation of cell-cell adhesion \| 1,00E-06 \| \| T cell activation \| 1,00E-06 \| \| T cell aggregation \| 1,00E-06 \| \| lymphocyte aggregation \| 1,00E-06 \| \| lymphocyte mediated immunity \| 1,10E-06 \| \| regulation of defense response \| 1,20E-06 \| \| leukocyte aggregation \| 1,20E-06 \| \| positive regulation of lymphocyte activation \| 2,00E-06 \| \| leukocyte cell-cell adhesion \| 2,00E-06 \| \| positive regulation of leukocyte chemotaxis \| 2,10E-06 \| \| positive regulation of adaptive immune response \| 2,70E-06 \| \| regulation of lymphocyte activation \| 3,20E-06 \| \| positive regulation of cell adhesion \| 3,50E-06 \| \| regulation of cell-cell adhesion \| 3,60E-06 \| \| homotypic cell-cell adhesion \| 4,30E-06 \| \| negative regulation of T cell activation \| 4,50E-06 \| \| lymphocyte proliferation \| 4,50E-06 \| \| positive regulation of leukocyte activation \| 4,60E-06 \| \| positive regulation of defense response \| 4,60E-06 \| \| mononuclear cell proliferation \| 4,70E-06 \| \| leukocyte chemotaxis \| 4,90E-06 \| \| regulation of leukocyte chemotaxis \| 5,00E-06 \| \| negative regulation of leukocyte cell-cell adhesion \| 5,90E-06 \| \| defense response to bacterium \| 6,10E-06 \| \| positive regulation of cell activation \| 6,60E-06 \| \| regulation of immune response \| 7,10E-06 \| \| cellular response to tumor necrosis factor \| 7,30E-06 \| \| T cell proliferation \| 7,80E-06 \| \| positive regulation of response to external stimulus \| 8,70E-06 \| \| positive regulation of immune response \| 9,20E-06 \| \| leukocyte activation \| 9,40E-06 \| \| negative regulation of homotypic cell-cell adhesion \| 9,90E-06 \| \| regulation of leukocyte activation \| 1,10E-05 \| \| regulation of innate immune response \| 1,20E-05 \| \| regulation of lymphocyte proliferation \| 1,40E-05 \| \| syncytium formation by plasma membrane fusion \| 1,40E-05 \| \| positive regulation of leukocyte migration \| 1,40E-05 \| \| regulation of mononuclear cell proliferation \| 1,50E-05 \| \| response to tumor necrosis factor \| 1,50E-05 \| \| syncytium formation \| 1,60E-05 \| \| lymphocyte activation \| 1,60E-05 \| \| negative regulation of lymphocyte activation \| 1,70E-05 \| \| regulation of leukocyte proliferation \| 1,80E-05 \| \| positive regulation of chemotaxis \| 1,80E-05 \| \| cellular response to interleukin-1 \| 1,90E-05 \| \| positive regulation of response to stimulus \| 2,00E-05 \| \| regulation of adaptive immune response \| 2,00E-05 \| \| regulation of cell activation \| 2,10E-05 \| \| lymphocyte migration \| 2,50E-05 \| |  |
| --- | --- | --- | --- | --- | --- | --- | --- | --- | --- | --- | --- | --- | --- | --- | --- | --- | --- | --- | --- | --- | --- | --- | --- | --- | --- | --- | --- | --- | --- | --- | --- | --- | --- | --- | --- | --- | --- | --- | --- | --- | --- | --- | --- | --- | --- | --- | --- | --- | --- | --- | --- | --- | --- | --- | --- | --- | --- | --- | --- | --- | --- | --- | --- | --- | --- | --- | --- | --- | --- | --- | --- | --- | --- | --- | --- | --- | --- | --- | --- | --- | --- | --- | --- | --- | --- | --- | --- | --- | --- | --- | --- | --- | --- | --- | --- | --- | --- | --- | --- | --- | --- | --- | --- | --- | --- | --- | --- | --- | --- | --- | --- | --- | --- | --- | --- | --- | --- | --- | --- | --- | --- | --- | --- | --- | --- | --- | --- | --- | --- | --- | --- | --- | --- | --- | --- | --- | --- | --- | --- | --- | --- | --- | --- | --- | --- |
